# Urachal carcinoma initially presenting only as a liver metastasis, a diagnostic and therapeutic challenge: first case in literature

**DOI:** 10.1097/MS9.0000000000002499

**Published:** 2024-08-26

**Authors:** George Bashour, George Hneino, Zain Aldin Zaher, Ali Dway, Georges Michael, Zuheir Alshehabi

**Affiliations:** a Cancer Research Center, Tishreen University Hospital; b Faculty of Medicine, Tishreen University; c Faculty of Medicine, Al-Andalus University, Tartus; d Department of Oncology, Tishreen University Hospital; e Department of Pathology, Tishreen University Hospital, Latakia, Syria

**Keywords:** adenocarcinoma, chemotherapy, liver metastasis, urachal carcinoma, urachus

## Abstract

**Introduction:**

Urachal carcinomas are uncommon malignant neoplasms comprising only 0.01% of all adult cancers. Most patients were aged from 58 to 64 years at diagnosis with 60 years being the median. It usually metastasizes to the lungs (22%), bones (22%), and liver (16%).

**Presentation:**

We report a case of a 71-year-old female patient who initially presented with two large liver masses and a small nodule on the anterior side of the bladder. The symptoms were nonspecific with abdominal discomfort. The final diagnosis of urachal adenocarcinoma was finalized with a biopsy of the bladder mass. The patient initially received six doses of FOLFOX6 without improvement and then Gem-Carbo, showing improvement after six doses. Finally, the patient received two doses of FOLFIRI-B with no response and kept deteriorating and died after 19 months of treatment.

**Discussion:**

About 90% of patients are symptomatic and hematuria is the most typical presenting symptom at diagnosis. The low incidence and the histopathologic similarities to adenocarcinoma from various sources pose a difficulty in recognizing the tumor. Our study presents the only case of a urachal carcinoma first manifesting with abdominal mass resulting from liver metastasis with no prior symptoms of urological origins. Also, our study presents the first attempt of using FLOFIRI-B to treat metastatic UraC.

**Conclusion:**

This case highlights the necessity for clinicopathological correlation to make the correct diagnosis and the challenges in the treatment which urges the need for further research to identify more effective treatment strategies for this rare cancer.

## Introduction

Highlights

- Urachal carcinoma is an uncommon tumor that rarely presents with metastasis without prior urological symptoms.
- Diagnosis is challenging due to its rarity and histopathologic similarities to other adenocarcinomas.
- Our case highlights the diagnostic and therapeutic challenges of urachal carcinoma.
- The diagnostic challenge was a presentation with the symptoms of a liver mass.
- The therapeutic challenge was highlighted with limited success despite multiple treatment regimens.
- Our case presents the first attempt to treat urachal carcinoma with FLOFIRI-B.

Urachal carcinomas (UraC) are uncommon malignant neoplasms comprising only 0.01% of all adult cancers^1^. The majority of urachal carcinomas are adenocarcinomas^1^. Males are 1.4 times more likely to have urachal tumors^1,2^. Most patients were aged from 58 to 64 years at diagnosis with 60 years being the median^3^. There may not always be an abdominal lump, although it is sometimes the initial and sole symptom^2^.

The majority of recent studies indicate a 5-year survival rate that ranges between 45 and 50% for invasive urachal carcinoma^4^.

It has a dismal prognosis due to the aggressive and late manifestation of the tumor^5^.

About 32–39% of patients experience metastatic disease, with 20–26% at the time of cystectomy and 39–48% during follow-up^4^. Distant metastasis is the first manifestation in about 21% of UraC patients^1^. Lungs (22%), bones (22%), liver (16%), lymph nodes (11%), and peritoneum (11%) are among the common locations of metastasis^2^.

There is not a standardized, efficient treatment for metastatic UraC yet^1^.

To our knowledge, this study presents the first documented UraC manifesting with abdominal mass resulting from liver metastasis as the only presentation with no prior symptoms of urological origins. This case report has been reported in line with the Surgical CAse Report (SCARE) Criteria and the first atempt to treat urachal carcinoma with FLOFIRI-B.^6^.

### Case presentation

**Figure 1:**
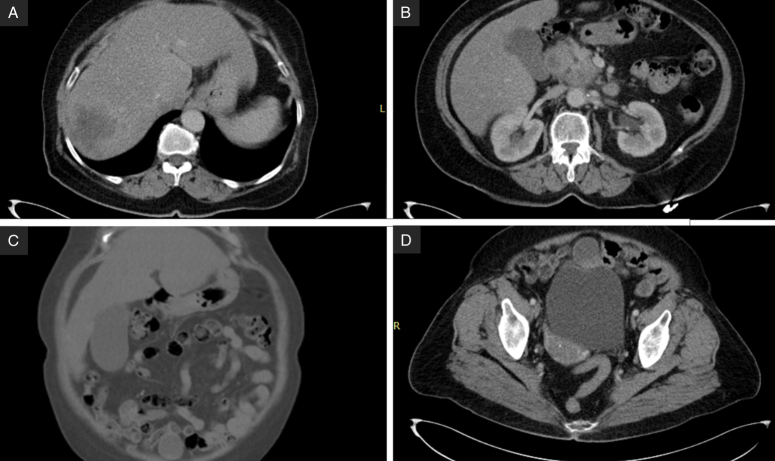
Computed Tomography scan, (A) Axial section: The seventh hepatic segment mass appearing hypodense, (B) Axial section: 53×77 mm mass in the hepatic hilum and infiltrating the head of the pancreas and duodenum, (C) coronal section: shows the hepatic helium mass, (D) Axial section: shows a hypodense nodule on the anterior wall of the bladder.

A 71-year-old female patient presented to the hospital with right upper quadrant (RUQ) discomfort and epigastric pain. She is a smoker with a history of treated type 2 diabetes and treated rheumatoid arthritis. Family history also included diffuse lymphoma in the patient’s eldest son. Clinical examination showed pallor, RUQ tenderness, and palpable hepatomegaly. An abdominal ultrasound (US) showed two separate hypoechoic masses in the liver: 60×75 mm in the seventh segment and 60×55 mm in the hilum. Abdominal and pelvic computed tomography (CT) showed 24 cm hepatomegaly, and a large mass infiltrating the head of the pancreas, duodenum, measuring 53×77 mm and extending to the liver hilum. It also showed a large asymmetrical hypodense lesion in the seventh segment (75×70 mm) with contrast enhancement and significant dilatation in the gallbladder without thickening in the wall and a 28×44 mm asymmetrical hyperperfused nodule adherent to the bladder (Fig. 1).

**Figure 2:**
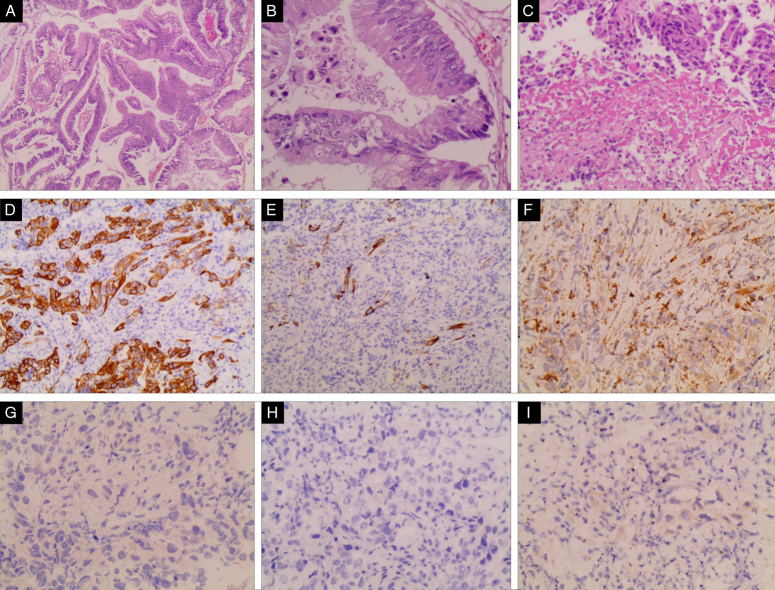
Histological examination: (A)(B) Urinary bladder biopsy H&E ×100 and ×200 in order, shows undifferentiated adenocarcinomas with histologic variations of mucinous and mixed morphology. (C) Liver lesion biopsy shows matching adenocarcinoma. (D)CK20 positive, (E) CK7 Negative, (F) GATA-3 Positive, (G) HEPAR-1 Negative, (H) PAX8 Negative, (I) TTF-1 Negative.

An ultrasound-guided biopsy of the liver was done. Pathological biopsy showed the involvement of poorly differentiated carcinoma in the liver. Upper and lower gastrointestinal endoscopies were performed to determine the initial source of the metastasis with no significant findings. Immunohistochemistry was positive for CK20, focally positive for CK7, and negative for GATA3, TTF-1, Pax-8, and Hep-Par 1 (Fig. 2).

A biomarkers panel was performed which included CA-125, CEA, AFP, and CA19-9, and were all normal. A cystoscopy and curettage were done to take a sample from the bladder’s dome. Pathological examination with the clinical and radiological findings confirmed the diagnosis of urachal adenocarcinoma as primary site of the patient liver metastatic carcinoma. EGFR mutation test showed wild type and PD-L1 was negative.

The patient received six doses of FOLFOX6 (folinic acid, 5-fluorouracil, and oxaliplatin) chemotherapy as the first-line treatment with no sign of improvement. As a result, the patient has been put on 6-doses of GemCarbo (gemcitabine and carboplatin) chemotherapy, which has proven effective, and the US showed a size decrease in the liver masses during this therapy. Later, the patient’s condition worsened and she developed ascites. She received two doses of FOLFIRI-B (folinic acid, fluorouracil, and irinotecan with bevacizumab) but did not get any response from it and was assigned to palliative care after 19 months of treatment in total and later died on the 20th month after the initial presentation.

## Discussion

Urachal carcinoma is a rare tumor that occurs in the bladder’s dome or anterior wall of the abdomen^1^. Ninety percent of patients are symptomatic, even though urachal cancer does not show any symptoms in its early stages^7^. Hematuria is the most typical presentation at diagnosis 58–82%^7^. Other less common signs and symptoms include abdominal pain or discomfort (14%), dysuria (12–14%), mucusuria ( 10%), pyuria, or generalized systemic symptoms such as nausea, fever, and weight loss^7^. In our case, there was no urological symptoms and the only presentation was the liver mass.

The low tumor incidence and the histopathologic characteristics of urachal carcinoma that match up with adenocarcinoma of various sources make the identification of urachal carcinoma challenging. This prompts the diagnosis by exclusion.

Evaluation of the tumor’s extent is done with CT scans. In most cases, it appears as a midline, supravesical, anterior mass extending into the anterior bladder dome^8^. An essential element in CT diagnostic imaging is the midline position of the mass, which is characteristic of urachal carcinoma^8^. MRI allows a better preoperative staging of urachal carcinomas^9^. Calcifications are pathognomonic for urachal adenocarcinoma in 50–70% of cases^10^.

Immunohistochemistry is crucial in the diagnosis of urachal carcinoma, with CK20 being mostly expressed^11^. In one study by Torenbeek *et al*.^12^ (1998), CEA was expressed in all cases of urachal adenocarcinoma, whereas vimentin, OC125, and HER-2/neu were not expressed. Some studies show that immunostains do not fully differentiate a urachal from colorectal carcinoma^13^. In our case, the liver mass IHC results had a differential diagnosis of either primary liver cholangiocarcinoma or metastatic adenocarcinoma from the pancreas or intestines which was a more consistent differential with the CT results.

Cystoscopy is required to determine whether cancer has reached the bladder’s urothelium, and a biopsy should also be carried out^10^. In our case, the cystoscopy was done to obtain a biopsy. It was done after the upper and lower gastrointestinal endoscopies as the small size of the mass on the bladder did not raise suspicion of being the primary tumor rather than a metastasis of the suspected intestinal tumor. The clinicopathological correlation between the CT signs of a midline anterior mass on the bladder dome and an adenocarcinoma finalized the diagnosis of UraC.

It is expected that there have not been extensive clinical investigations to identify treatments for UraC due to the low frequency of the disease and most of the data are accessible from case reports utilizing a variety of chemotherapy regimens. In a study of 17 UraC by Chen *et al*.^14^ (2014), the overall survival (OS) was 4.8 years across all patients and the OS in patients who were eligible for surgery was 6.2 years, but many urachal carcinoma patients present with advanced diseases that cannot be surgically cured and the median survival was 1.8 years.

GemCarbo is the recommended first-line for metastatic urinary tract carcinoma in patients who have cisplatin contraindicated^5^. Despite GemCarbo patients having worse baseline characteristics, patients treated with GemCis (gemcitabine and cisplatin) did not have a better overall survival rate^15^. FOLFIRI-B is an effective first-line treatment in advanced colorectal carcinoma (CRC) and with the histopathological similarities it has been suggested as a potential treatment for UraC^16,17^. Unfortunately, there has been only one paper using FLOFIRI treatment for metastatic UraC in six patients with modest results^17^. We could not find any evidence in the literature of using FLOFIRI-B in UraC.

Our patient started the treatment with FOLFOX6 and showed no improvement after six doses. The second line was GemCarbo (gemcitabine & carboplatin) instead of GemCis as the patient is >70 years old, cisplatin had to be swapped for carboplatin. The patient showed improvement after taking six doses of GemCarbo. Then she received two doses of FOLFIRI-B as a final line treatment after developing ascites but did not get any response from it and the tumor kept progressing. Our study presents the first attempt of using FLOFIRI-B to treat metastatic UraC.

There is an urgent need to create systemic medicines with better results for those with recurring or metastatic urachal cancer.

## Conclusion

We report a unique case of urachal carcinoma that highlights the clinical and diagnostic challenges as it only manifested as a metastasis to the liver with no urological symptoms. This case highlights the necessity for clinicopathological correlation to make the correct diagnosis and the need for a standardized, efficient treatment for metastatic UraC as this case explored multiple therapeutic regimens with unfortunate results.

## Ethical approval

Not applicable.

## Consent

Written informed consent was obtained from the patient for publication of this case report and accompanying images. A copy of the written consent is available for review by the Editor-in-Chief of this journal on request.

## Source of funding

Not applicable.

## Author contribution

All authors contributed to the writing and editing of this manuscript. G.B., G.H., and Z.A.Z.: collecting references, original draft, and writing and editing; A.D.: original draft writing; M.G.: reviewing and supervision; Z.A.: final reviewing and supervision.

## Conflicts of interest disclosure

The authors declare no conflicts of interest.

## Research registration unique identifying number (UIN)

Not applicable.

## Guarantor

Zuheir Alshehabi.

## Data availability statement

Not applicable.

## Provenance and peer review

Not commissioned, externally peer-reviewed.
